# Biological sex representation and reporting in stereotactic body radiotherapy for kidney cancer: A review of clinical studies

**DOI:** 10.1016/j.ctro.2025.101034

**Published:** 2025-08-18

**Authors:** Sylvia Nwokolo, Laure Marignol

**Affiliations:** Trinity St. James’s Cancer Institute, Radiobiology and Molecular Oncology Research Group, Applied Radiation Therapy Trinity, Discipline of Radiation Therapy, Trinity College Dublin, Ireland

**Keywords:** Biological sex, Kidney cancer, SBRT

## Abstract

**Background:**

Stereotactic body radiotherapy (SBRT) is evolving as treatment modality for kidney cancer alongside increased recognition of the importance of sex as a biological variable (SABV) policy in generating deeper insights.

**Purpose:**

This review aimed to assess the representation of male and female patients and the reporting of sex-specific outcomes in SBRT clinical studies for primary localised kidney cancer.

**Methodology:**

A database search with appropriate search terms was carried out on EMBASE, CINAHL, and Web of Science. Consideration of biological sex was assessed with the male: female ratio, reporting of sex-specific treatment outcomes, and inclusion of sex as a variable in univariate and/or multivariate analysis.

***Main findings***:

Twenty-two studies were included. Of 961 patients (with sex stated), 29.24 % were females, with a mean male to female ratio of 2.57 and standard deviation of 1.22 across the studies. There was no relationship between the year, region and study type, and the male to female ratio recorded (Chi-squared test, p = 0.099, 0.29, and 0.719 respectively). Only six studies were sex inclusive, with three reporting sex-based outcomes, and three including sex-specific analysis. Of these, only one study reported significant difference in survival.

**Conclusion:**

Supporting the integration of SABV will promote more equitable and biologically informed evidence for the use of SBRT in the management of kidney cancer.

## Highlights

• SBRT is gaining traction for localized kidney cancer, presenting an opportunity to align its clinical evidence base with SABV policy.
• SABV is an evolving norm that research communities are integrating at different rates.
• One study reported sex-based survival differences, highlighting the need to integrate SABV for clinical insights.
• Support for SABV integration in SBRT analysis and reporting will foster equitable and biologically informed kidney cancer evidence.

## Introduction

Mounting evidence identifies sex disparities in incidence and outcome of kidney cancer [[1], [2], [3], [4]]. Female sex is associated with significantly smaller tumours, lower pathological grade at diagnosis, and lower incidence of local and distant metastases, compared to males [1]. This disparity was seen across age, time-period, and race [5]. The 2:1 male to female ratio remains stable despite the reported levelling of exposures and risk factors such as smoking and obesity between the sexes [2,5]. Localised disease has primarily been treated by surgery, with radiation therapy (RT) historically playing a small role owing to its intrinsically radioresistant nature [6,7]. Increasingly, stereotactic body radiation therapy (SBRT) is proving a safe non-invasive ablative approach that can achieve local control and could benefit inoperable patients [8]. Reports that biological sex could influence the radiation response [[9], [10], [11]] indicate that the consideration of sex as a biological variable could inform treatment optimisations [12,13].

Sex as a Biological Variable (SABV) policy was introduced to encourage the inclusion of women and minorities in clinical research, historically prevented [[14], [15], [16]]. This policy mainly relates to “sex”, the biological and physiological characteristics of males and females, as opposed to “gender” which refers to the “socially constructed characteristics of women, men, girls and boys” [17]. The importance of SABV is being closely examined, as growing evidence shows that sex differences extend beyond reproductive and hormonal factors to include cellular, molecular, and genetic elements [18]. Lack of adherence is linked to female underrepresentation, relative to disease prevalence [19,20].

Genetic and molecular differences primarily can be attributed to functional differences of gene expression in the X and Y chromosomes, which is independent of the sex hormones [13]. In kidney tumours, higher expression of the X-chromosome in women, and increased ESCAPE genes (that escape X-chromosome inactivation) was reported, some of which are tumour suppressive EXIT genes [1,2]. Female specific immune mechanisms also make up molecular contribution to this sex-disparity [2]. In males, this genetic variation is mainly attributed to the mosaic loss of Y chromosome (LoY), which leads to difference in mutation frequency of oncogenes and tumour suppressor genes, and is associated with various cancers, including kidney cancer [[21], [22], [23]]. Sex-specific microRNA radiation response has also been identified in preclinical studies of male and female mice [24,25], with the female sex associated with increased radiosensitivity in clinical studies [12,26,27].

Increase in the utilisation of SBRT for localised kidney cancer calls for treatment optimisation [6]. In the era of precision medicine and stratification of patients into the most biologically appropriate groups to optimise therapeutic response [28], the appropriate consideration of biological sex can be anticipated to help address disease heterogeneity and its impact on treatment effectiveness, alongside other genetic, metabolic, and physiologic factors such as age, ethnicity, and tumour grade [19,29]. This study aims to assess the consideration of biological sex in clinical studies on SBRT for primary localised kidney cancer.

## Methodology

### Search strategy for identification of studies

**Fig. 1:**
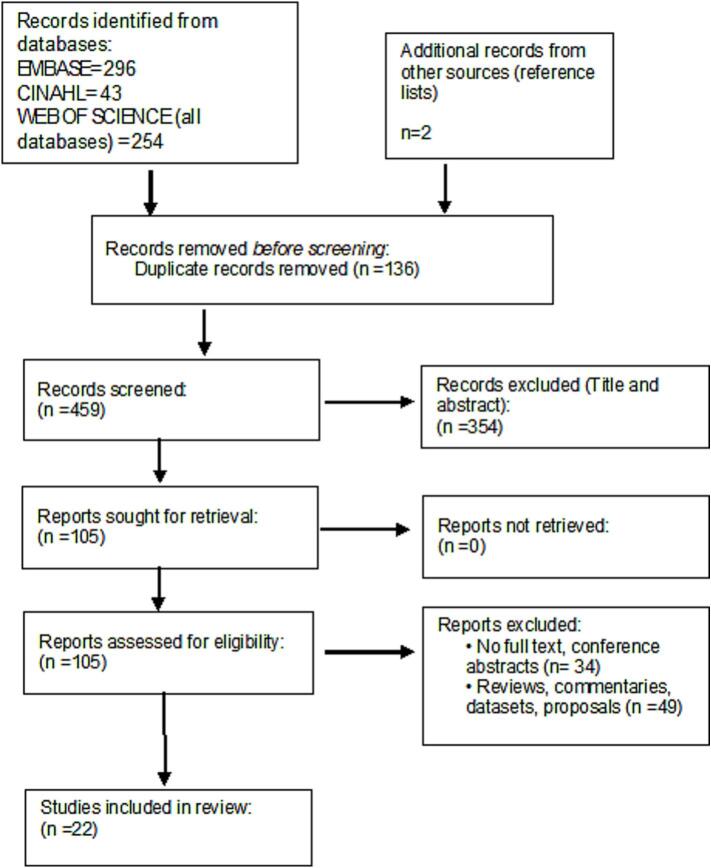
Prisma flow diagram showing each screening process, leading up to the final studies included for data extraction.

The Preferred Reporting Items for Systematic reviews and Meta-Analyses extension for Scoping Reviews (PRISMA-ScR) checklist was adhered to. The final search was conducted on 30/12/2024. The following databases were used; EMBASE, CINAHL, and Web of Science. The following search terms were applied, ((kidney OR renal) NEAR/3 (cancer OR tumour OR tumor OR carcinoma OR neoplasm)) AND (‘stereotactic body radiation therapy’ OR (‘SABR’ OR ‘SABRT’ OR ‘SBRT’) OR (stereotactic NEAR/3 (‘radiation therapy’ OR ‘radiotherapy’))) AND (‘clinical trial’ OR ‘randomised controlled trial’). The full search strategy is reported in appendix A . As SBRT in the management of kidney cancer is a recent application, a date restriction of 2014–2024 was also applied. The reference manager Endnote was used in the screening of the identified records. The first screening was conducted to eliminate duplicate records. This was followed by a title and abstract screening, and finally a full text assessment. Additional relevant records were also included from reference lists. The identification and screening of the records are summarised in the PRISMA diagram in Fig. 1.

### Type of studies

Only peer-reviewed records in English were included. Randomised and non-randomised trials, and cohort studies were included. Retrospective and Phase I feasibility studies were included. Conference abstracts, abstract-only texts and commentaries were excluded. Systematic and literature reviews were excluded. Individual participant data meta-analyses were included.

### Type of participants

Male and female patients with primary kidney cancer were included, and oligometastatic kidney cancer was excluded. Bilateral primary kidney cancer patients were included.

### Type of Interventions

Only studies that investigate stereotactic body radiotherapy were included. Studies only investigating surgery or other ablative therapies such as cryotherapy and radiofrequency ablation were not included. Comparative studies investigating SBRT and any other treatment management for primary kidney cancer were included. All SBRT prescription dose or fractionation schedules were included.

### Type of outcomes

The following variables were extracted from each article: [1] the author, [2] the year of publication, [3] the country, [4] the type of study, [5] the number of male and female participants included, [6] the tumour stage included, [7] the prescription dose and fractionation, [8] the inclusion of sex- specific treatment outcomes (i.e., reporting treatment outcomes for male and female participants separately), and [9] the inclusion of sex as a variable in data analysis. Studies were classified as sex-inclusive if sex-specific outcomes were reported, or sex was included as a variable in univariate and/or multivariate data analysis.

### Data analysis

The distribution of male and female participants was reported as M:F ratio and percentages. The range, mean, and standard deviation of these ratios were calculated to measure the variability across the studies. The distribution of studies in terms of their recorded M: F (a ratio of 1, less than 1, greater than 1, and greater than 5) was reported as percentages. The study and treatment characteristics assessed include the year (2014–2018, and 2019–2024), region (Europe, North America, Australia, Asia), study type (prospective or retrospective), prescription dose and fractionation. The distribution was reported as percentages, and the Chi-squared test of independence used to assess the influence of these factors on sex consideration. The inclusion of sex-based data and analysis were also reported as percentages.

### Description of included studies

A total of 595 articles were screened, of which 136 duplicates were excluded, and following the title and abstract screening, and full text review, 22 studies were included. Fourteen (63.64 %) were prospective studies, while eight (36.36 %) were retrospective, two of which were individual participant data meta-analysis from the same institutions [30,31]. Five studies (22.7 %) were based in Europe, eight (36.36 %) were based in North America, four (18.2 %) were based in Asia, two (9.1 %) were based in Australia, while three (13.64 %) were from international institutions. Most of the included studies were conducted to investigate tumour control, survival, and treatment related toxicities, including renal function which was mainly assessed using the Glomerular Filtration Rate (GFR). Three studies investigated the feasibility and/or safety of the treatment delivery [[32], [33], [34]], and four studies only examined the side effects of treatment delivery [31,[35], [36], [37]]. Swaminath et al. [44] was the only study conducted to assess patient reported quality of life (QOL) following SBRT. Zhao et al. [37], investigated SBRT for upper tract urothelial carcinoma, including RCC. Two of the studies investigated the use of neoadjuvant SBRT for patients with RCC and inferior vena-cava tumour thrombus (IVC-TT) [34,38], while the remaining studies explored definitive SBRT for kidney cancer. Two comparative studies were also included. Grant et al. [46] investigated the use of surgery, tumour ablation, SBRT, or observation in patients with early-stage kidney cancer, while Tan et al. [31], explored long-term renal function outcomes following SBRT to solitary versus bilateral kidney tumour. The mean sample size across the studies was 44, with a range of 6–190.

There was heterogeneity across and within the studies in terms of tumour stage and dose fractionation adopted, with inconsistency in the data reporting for tumour stage. Only fifteen studies used the TNM staging in their report of patient characteristics. The remaining studies recorded this in the form of tumour length and/or volume (Table 1). Zhao et al. [37], was the only study which did not report any patient characteristics including sex, age, race, and tumour stage, and was therefore excluded from the statistical analysis. Across the studies which reported the TNM staging, the included stage ranged from T1-T4. In terms of dose, a total dose range of 25 Gy-70 Gy was observed, with fractionation ranging from single to 10 fraction schedules. Most of the studies included more than one fractionation schedule, with only four studies adopting a single schedule (Table 1).

**Table 1:** Characteristics and sex inclusion of the included studies. Abbreviations: TNM (Tumour, Node, Metastases) AUS (Australia), NL (Netherlands), CAN (Canada), GE (Germany), JP (Japan), PRS (Prospective), RTS (Retrospective), Gy (Gray), # (Fractions).

Study	Year	Region	Study type	No. of patients	No. (M,F) (M:F)	TNM stage	Fractionation	Sex inclusion
Funayama et al.	2019	Japan	PRS	13	10, 3 (3.33)	T1	60/70 Gy/10#	Yes
Glicksman et al.	2023	Canada	PRS	74	49, 25 (1.96)	T1-4	30–45 Gy/5# Or 42 Gy/3#	No
Hannan et al.	2023	USA	PRS	16	11, 5 (2.2)	T1	36 Gy/3# Or 40 Gy/5#	No
Rivas et al.	2024	Spain	RTS	23	14, 9 (1.55)	−	40–64 Gy/3–8#	No
Siva et al.	2024	AUS and NL	PRS	70	49, 21 (2.33)	T1-3	Single 26 Gy Or 42 Gy/3#	No
Yamamoto et al.	2021	Japan	RTS	29	22, 7 (3.14)	T1	50/60/70 Gy/10#	Yes
Yim et al.	2024	USA	PRS	20	13, 7 (1.9)	T1-3	40 Gy/5#	No
Zakar et al.	2023	UK	PRS	19	9, 10 (0.9)	−	Single 26 Gy Or 42 Gy/3#	No
Chang et al.	2016	Canada	RTS	16	11, 5 (2.2)	T1-3	30–40 Gy/5#	No
Pham et al.	2014	NL	PRS	20	15, 5 (3)	−	Single 26 Gy Or 42 Gy/3#	No
Ponsky et al.	2015	USA	PRS	19	13, 6 (2.17)	T1-3	24–48 Gy/4#	No
Siva et al.	2016	AUS	PRS	21	14, 7 (2)	T1-3	Single 26 Gy Or 42 Gy/3#	No
Siva et al.	2017	AUS	PRS	37	28, 9 (3.11)	T1-2	Single 26 Gy Or 42 Gy/3#	No
Yamamoto et al.	2016	Japan	RTS	14	11, 3 (3.67)	−	50/60/70/10#	Yes
Chen et al.	2024	China	PRS	8	6, 2 (3)	T1-4	30 Gy/5#	Yes
Margulis et al.	2021	USA	PRS	6	3, 3 (1)	T1-4	40 Gy/5#	Yes
Siva et al.	2022	AUS, CAN, GE, JP, USA.	Meta-analysis (RTS)	190	139, 51 (2.7)	−	Single 25 Gy Or 35–48 Gy/3–5#	No
Swaminath et al.	2021	Canada	PRS	32	22, 10 (2.2)	T1	30–45 Gy/5# Or 42 Gy/3#	No
Staehler et al.	2015	Germany	PRS	40	35, 5 (7)	−	Single 25y	No
Grant et al.	2020	USA	RTS	104 (SBRT) 200,839 (Total)	67, 37 (1.8)	T1	40 Gy/5# Or 48 Gy/3#	Yes
Tan et al.	2024	AUS, CAN, GE, JP, USA.	Meta-analysis (RTS)	190	139, 51 (2.7)	T1-2	25–35 Gy/1–3#	No
Zhao et al.	2024	UK	RTS	6 (Kidney) 34 (Total)	−	−	36/39/42 Gy/3#	No

### Female representation and sex-based reporting

Across the twenty-two studies, 967 patients were included, with the patient sex disclosed in twenty-one studies (95.45 %). Of these, 680 were male (70.76 %) and 281 were female (29.24 %). A mean male to female ratio of 2.57 was calculated with a standard deviation of 1.22 and range of 0.9–7 across the studies. Nineteen studies (90.48 %) recorded more male participants compared to females. One study (4.76 %) had a male to female ratio less than 1 [39], one study (4.76 %) had an equal number [38], eighteen studies (85.71 %) had a ratio between 1 and 5 [[30], [31], [32], [33], [34], [35], [36],[40], [41], [42], [43], [44], [45], [46], [47], [48], [49], [50]], while one (4.67 %) had a ratio greater than 5 [51]. There was no relationship between the year, region and type of study, and the male to female ratio observed (Chi-squared test, p = 0.099, 0.29, and 0.719 respectively). Three studies were excluded from the Chi-squared test for the region, as they were international studies [30,31,44]. Test of independence was not conducted for treatment dose and fractionation as this was quite varied across and within the studies.

**Fig. 2:**
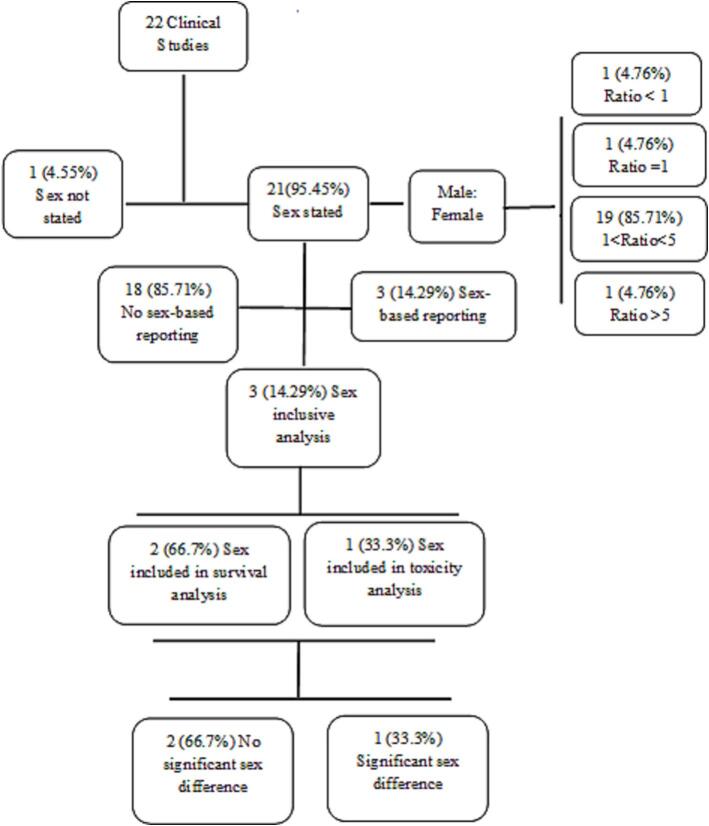
Box diagram of the included studies, and distribution according to sex inclusion.

Six articles (28.57 %) were sex inclusive [34,36,38,40,45,50] (Fig. 2). Three of these (50 %) included sex-based reporting, and the other three (50 %) included sex as a variable in data analysis. All the articles that included sex-based data reporting recorded a low number of participants (range of 6–13) (Table 1) which enabled sex inclusion, as the data was reported on an individual basis with sex as a variable. Sex was included as a variable in the reporting of tumour characteristics for all three studies. Funayama et al. [40], included sex-based reporting for tumour response, chronic kidney disease (CKD), and initiation of haemodialysis. Chen et al [34], reported the length and widest diameter of IVC-TT before and after SBRT with sex as a variable, while Margulis et al. [38], reported tumour progression and immunochemistry following SBRT. The studies which reported sex-based data however did not include sex as a variable in their analysis.

Of the twenty-one studies which include age as a patient characteristic, three of these included age in the data reporting, all of which also included sex-based reporting, while five studies included age as a variable in analysis, three of which also included sex in their analysis. Only four studies reported race as a variable, with a majority of white participants recorded (range of 56 %-90 %). Grant et al. [46] was the only study which included sex, age and race in their analysis.

### Inclusion of sex as a variable in analysis

Three studies (14.29 %) included sex as a variable in their data analysis. This was recorded in univariate or multivariate analysis. Two of these studies were by the same first author who investigated the survival outcomes following SBRT for kidney cancer and included univariate analysis. The first study was conducted to investigate renal atrophy, with sex-specific analysis for Mann-Whitney test between post-treatment irradiated kidney volume [36]. The more recent study by Yamamoto et al. further explored tumour control, survival and adverse effects, with the inclusion of sex as a variable in log-rank tests for overall survival and progression free survival [45]. There was no significant difference between the male and female outcomes measured in both studies (p = 0.31 and 0.19 respectively). Grant et al. [50], conducted a comparative study on the use of surgery, tumour ablation (cryotherapy or thermal ablation) and SBRT for patients with stage I kidney cancer. Sex was included as a variable in the multivariate Cox proportional hazards regression for overall survival (OS) with propensity score adjustments. A hazard ratio of 1 was reported for the female population, while a hazard ratio of 1.16, p < 0.001 was recorded for the male cohort, which indicates worsened survival. However, this was conducted for the entire population as opposed to separate analysis for each treatment modality.

## Discussion

Supporting researchers in integrating SABV into the design, analysis, and interpretation of studies will enrich the scientific rigor and clinical relevance of radiation therapy innovations. This review highlights that SABV is an evolving norm that research communities are integrating at different rates. This is evidenced by underrepresentation of female participants, and lack of both sex-specific reported outcomes, and sex-based data analysis in most of the studies reviewed in this analysis.

First, inclusion of SABV should begin at the “scientific premise of the proposed research”, therefore the literature review, as the research question(s) can be influenced by sex differences [18]. For most cancer sites, the existing evidence for sex differences associates the male sex with poorer prognosis and outcomes but reduced adverse effects. In terms of radiation therapy, females have more radio-sensitive genes compared to males, which may also increase their risk of developing radiation-induced toxicities [12,52]. In a pooled analysis study for esophagogastric cancer, it was found that females received less chemotherapy, with a significantly higher proportion only getting 1–3 rounds compared to men, due to a greater risk of developing gastro-intestinal toxicities [53]. This is also reflected in kidney cancer [54,55], as the female sex was indicated as a predictive factor for severe toxicity following Sunitinib for metastatic RCC [4]. Despite the available evidence, the issue of sex difference was not addressed in the studies reviewed, highlighting an important opportunity for future research.

Second, the implementation of a “rigorous experimental design for robust and unbiased results” is recommended [18]. In the application of SABV, this is reflected in the recruitment of a sufficient number of males and females needed to adequately power the detection of sex differences [56,57]. Of the 961 patients included in this review (of which sex was stated), there were 680 males (70.76 %) and 281 females (29.24 %). The mean M: F across the twenty-one studies was 2.57, with nineteen of the studies (90.5 %) having a ratio greater than 1. These results are similar to that of a recent review conducted to assess sex inclusion in preclinical and clinical trials for kidney cancer [58]. This review obtained a mean M: F of 2.3, with 96 % of the studies having a ratio greater than 1. This limited female inclusion may be attributed to the known 2:1 male to female ratio for the prevalence of kidney cancer [2,5]. Balanced recruitment, with sufficient inclusion of both sexes to enable adequately powered sex-based analyses, should be considered alongside maintaining representativeness of the disease prevalence.

Third, “Consideration of relevant biological variables” such as sex, age, and race should be integrated in medical research [18]. Sex consideration can be achieved by sex-specific data reporting and sex-based analysis. Of the studies which disclose patient sex in this review, six studies (28.57 %) were considered sex inclusive. Three of these (14.29 %) reported sex-specific outcomes, but did not consider sex in their analysis, while three studies (14.29 %) included sex-based univariate or multivariate analysis. Low sex-specific outcome reporting of 4 % was also observed in the previously mentioned review [58], however a greater proportion of the studies (37 %) included sex as a controlled variable in analysis. This lack of sex-based analysis could be attributed to the small sample sizes included. Across the twenty-two studies, the mean number of patients was 44, with a range of 6–190. These small sample sizes likely reflect the low proportion of patients with primary kidney cancer receiving SBRT [6], suggesting that collaborative networks are likely needed to support the integration of SABV in this patient population. Of the studies that conducted sex-based analysis, only one recorded significant reduction in survival in the male cohort [50], adding to existing evidence in kidney cancer therapy that female patients tend to have better survival outcomes [4,55].

Finally, SABV highlights the “authentication of key biological and/or chemical resources” [18]. This refers to the validation of biological sex in humans with a karyotype, therefore the presence of XX or XY chromosomes. Evidence suggests that the bias effect of these chromosomes, such as the oncogenic activity of the Y chromosome and tumour suppressor activity of the X chromosome contribute to sex differences in carcinogenesis [23,59,60]. For instance, the loss of Y chromosome specific genes which has been linked to various cancer types including RCC [21,23,61], radio-resistance in non-small cell lung cancer [62], and immune response in bladder cancer [63]. Developing tools to investigate these chromosomal differences will accelerate our understanding of sex-specific disease mechanisms and the advancement of tailored therapeutic strategies. This work will complement ongoing efforts to examine the full range of biological sex-related factors that may influence cancer outcomes. Hormonal factors, such as the protective effects of oestrogens in colorectal cancer,[64] sex-specific immune responses that contribute to differences in melanoma progression and immunotherapy efficacy,[65] and metabolic differences affecting drug pharmacokinetics,[66] are some recognised examples.

## Conclusion

Cultivating the integration of SABV in the development of SBRT for male and female patients with kidney cancer will promote more equitable and biologically informed evidence.

## Declaration of competing interest

The authors declare that they have no known competing financial interests or personal relationships that could have appeared to influence the work reported in this paper.

## References

[1] Peired A.J., Campi R., Angelotti M.L., Antonelli G., Conte C., Lazzeri E. (2021). Sex and gender differences in kidney cancer: clinical and experimental evidence. Cancers (Basel).

[2] Laskar R.S., Li P., Ecsedi S., Abedi-Ardekani B., Durand G., Robinot N. (2021). Sexual dimorphism in cancer: insights from transcriptional signatures in kidney tissue and renal cell carcinoma. Hum Mol Genet.

[3] Mamtani R., Wang X.V., Gyawali B., DiPaola R.S., Epperson C.N., Haas N.B. (2019). Association between age and sex and mortality after adjuvant therapy for renal cancer. Cancer.

[4] van der Veldt A.A., Boven E., Helgason H.H., van Wouwe M., Berkhof J., de Gast G. (2008). Predictive factors for severe toxicity of sunitinib in unselected patients with advanced renal cell cancer. Br J Cancer.

[5] Scelo G., Li P., Chanudet E., Muller D.C. (2018). Variability of sex disparities in cancer incidence over 30 years: the striking case of kidney cancer. Eur Urol Focus.

[6] Haque W., Verma V., Lewis G.D., Lo S.S., Butler E.B., Teh B.S. (2018). Utilization of radiotherapy and stereotactic body radiation therapy for renal cell cancer in the USA. Future Oncol.

[7] Blanco A.I., Teh B.S., Amato R.J. (2011). Role of radiation therapy in the management of renal cell cancer. Cancers (Basel).

[8] Grant S., Lei X., Hess K., Smith G.L., Matin S., Wood C. (2019). Stereotactic body radiotherapy for the definitive treatment of early stage kidney cancer: a survival comparison with surgery, tumor ablation, and observation. Int J Radiat Oncol Biol Phys.

[9] Meunier A., Marignol L. (2020). The radiotherapy cancer patient: female inclusive, but male dominated. Int J Radiat Biol.

[10] Besplug J., Burke P., Ponton A., Filkowski J., Titov V., Kovalchuk I. (2005). Sex and tissue-specific differences in low-dose radiation-induced oncogenic signaling. Int J Radiat Biol.

[11] Sui F., Sun W., Su X., Chen P., Hou P., Shi B. (2018). Gender-related differences in the association between concomitant amplification of AIB1 and HER2 and clinical outcomes in glioma patients. Pathology - Research and Practice.

[12] De Courcy L., Bezak E., Marcu L.G. (2020). Gender-dependent radiotherapy: the next step in personalised medicine?. Crit Rev Oncol Hematol.

[13] Mauvais-Jarvis F., Bairey Merz N., Barnes P.J., Brinton R.D., Carrero J.J., DeMeo D.L. (2020). Sex and gender: modifiers of health, disease, and medicine. Lancet.

[14] Arnegard M.E., Whitten L.A., Hunter C., Clayton J.A. (2020). Sex as a biological variable: a 5-year progress report and call to action. J Womens Health (Larchmt).

[15] Clayton J.A. (2016). Studying both sexes: a guiding principle for biomedicine. FASEB J.

[16] Heidari S., Fernandez D.G.E., Coates A., Hosseinpoor A.R., Asma S., Farrar J. (2024). WHO's adoption of SAGER guidelines and GATHER: setting standards for better science with sex and gender in mind. Lancet.

[17] Peters S.A.E., Woodward M. (2023). A roadmap for sex- and gender-disaggregated health research. BMC Med.

[18] Clayton J.A. (2018). Applying the new SABV (sex as a biological variable) policy to research and clinical care. Physiol Behav.

[19] Feldman S., Ammar W., Lo K., Trepman E., van Zuylen M., Etzioni O. (2019). Quantifying sex bias in clinical studies at scale with automated data extraction. JAMA Netw Open.

[20] Geller S.E., Koch A.R., Roesch P., Filut A., Hallgren E., Carnes M. (2018). The more things change, the more they stay the same: a study to evaluate compliance with inclusion and assessment of women and minorities in randomized controlled trials. Acad Med.

[21] Gutiérrez-Hurtado I.A., Sánchez-Méndez A.D., Becerra-Loaiza D.S., Rangel-Villalobos H., Torres-Carrillo N., Gallegos-Arreola M.P. (2024). Loss of the Y chromosome: a review of molecular mechanisms, age inference, and implications for Men's health. Int J Mol Sci.

[22] Russo P., Bizzarri F.P., Filomena G.B., Marino F., Iacovelli R., Ciccarese C. (2024). Relationship between loss of Y chromosome and urologic cancers: new future perspectives. Cancers (Basel).

[23] Minner S., Kilgué A., Stahl P., Weikert S., Rink M., Dahlem R. (2010). Y chromosome loss is a frequent early event in urothelial bladder cancer. Pathology.

[24] Koturbash I., Zemp F., Kolb B., Kovalchuk O. (2011). Sex-specific radiation-induced microRNAome responses in the hippocampus, cerebellum and frontal cortex in a mouse model. Mutation Research/genetic Toxicology and Environmental Mutagenesis.

[25] Ilnytskyy Y., Zemp F.J., Koturbash I., Kovalchuk O. (2008). Altered microRNA expression patterns in irradiated hematopoietic tissues suggest a sex-specific protective mechanism. Biochem Biophys Res Commun.

[26] Panian J., Lin X., Simantov R., Derweesh I., Choueiri T.K., McKay R.R. (2020). The impact of age and gender on outcomes of patients with advanced renal cell carcinoma treated with targeted therapy. Clin Genitourin Cancer.

[27] Luo H.-S., Xu H.-Y., Du Z.-S., Li X.-Y., Wu S.-X., Huang H.-C. (2019). Impact of sex on the prognosis of patients with esophageal squamous cell cancer underwent definitive radiotherapy: a propensity score-matched analysis. Radiat Oncol.

[28] Day S., Coombes R.C., McGrath-Lone L., Schoenborn C., Ward H. (2017). Stratified, precision or personalised medicine? cancer services in the ‘real world’ of a London hospital. Sociol Health Illn.

[29] Krzyszczyk P., Acevedo A., Davidoff E.J., Timmins L.M., Marrero-Berrios I., Patel M. (2018). The growing role of precision and personalized medicine for cancer treatment. Technology (Singap World Sci).

[30] Siva S., Ali M., Correa R.J.M., Muacevic A., Ponsky L., Ellis R.J. (2022). 5-year outcomes after stereotactic ablative body radiotherapy for primary renal cell carcinoma: an individual patient data meta-analysis from IROCK (the International Radiosurgery Consortium of the Kidney). Lancet Oncol.

[31] Tan V.S., Correa R.J.M., Warner A., Ali M., Muacevic A., Ponsky L. (2024). Long-term renal function outcomes after stereotactic ablative body radiotherapy for primary renal cell carcinoma including patients with a solitary kidney: a report from the international radiosurgery oncology consortium of the kidney. Eur UrolOncology.

[32] Pham D., Thompson A., Kron T., Foroudi F., Kolsky M.S., Devereux T. (2014). Stereotactic ablative body radiation therapy for primary kidney cancer: a 3-dimensional conformal technique associated with low rates of early toxicity. Int J Radiat Oncol Biol Phys.

[33] Siva S., Pham D., Kron T., Bressel M., Lam J., Tan T.H. (2017). Stereotactic ablative body radiotherapy for inoperable primary kidney cancer: a prospective clinical trial. BJU Int.

[34] Chen J., Liu Z., Peng R., Liu Y., Zhang H., Wang G. (2024). Neoadjuvant stereotactic ablative body radiotherapy combined with surgical treatment for renal cell carcinoma and inferior vena cava tumor thrombus: a prospective pilot study. Bmc Urology.

[35] Siva S., Jackson P., Kron T., Bressel M., Lau E., Hofman M. (2016). Impact of stereotactic radiotherapy on kidney function in primary renal cell carcinoma: establishing a dose-response relationship. Radiother Oncol.

[36] Yamamoto T., Kadoya N., Takeda K., Matsushita H., Umezawa R., Sato K. (2016). Renal atrophy after stereotactic body radiotherapy for renal cell carcinoma. Radiat Oncol.

[37] Zhao Y., Cozma A., Ding Y., Perles L.A., Reiazi R., Chen X. (2024). Upper urinary tract stereotactic body radiotherapy using a 1.5 tesla magnetic resonance imaging-guided linear accelerator: workflow and physics considerations. Cancers.

[38] Margulis V., Freifeld Y., Pop L.M., Manna S., Kapur P., Pedrosa I. (2021). Neoadjuvant SABR for renal cell carcinoma inferior vena cava tumor thrombus-safety lead-in results of a phase 2 trial. Int J Radiat Oncol Biol Phys.

[39] Zarkar A., Henderson D., Carver A., Heyes G., Harrop V., Tutill S. (2023). First UK patient cohort treated with stereotactic ablative radiotherapy for primary kidney cancer. Bjui Compass.

[40] Funayama S., Onishi H., Kuriyama K., Komiyama T., Marino K., Araya M. (2019). Renal cancer is not radioresistant: Slowly but continuing shrinkage of the tumor after stereotactic body radiation therapy. Technology in Cancer Research and Treatment.

[41] Glicksman R.M., Cheung P., Korol R., Niglas M., Nusrat H., Erler D. (2023). Stereotactic body radiotherapy for renal cell carcinoma: oncological and renal function outcomes. Clin Oncol.

[42] Hannan R., McLaughlin M.F., Pop L.M., Pedrosa I., Kapur P., Garant A. (2023). phase 2 trial of stereotactic ablative radiotherapy for patients with renal cancer. Eur Urol.

[43] Rivas D., de la Torre-Luque A., Moreno-Olmedo E., Moreno P., Suárez V., Serradilla A. (2024). Stereotactic body radiotherapy: is less fractionation more effective in adrenal and renal malignant lesions?. World J Urol.

[44] Siva S., Bressel M., Sidhom M., Sridharan S., Vanneste B.G.L., Davey R. (2024). Stereotactic ablative body radiotherapy for primary kidney cancer (TROG 15.03 FASTRACK II): a non-randomised phase 2 trial. Lancet Oncol.

[45] Yamamoto T., Kawasaki Y., Umezawa R., Kadoya N., Matsushita H., Takeda K. (2021). Stereotactic body radiotherapy for kidney cancer: a 10-year experience from a single institute. J Radiat Res.

[46] Yim K., Hsu S.-H., Nolazco J.I., Cagney D., Mak R.H., D'Andrea V. (2024). Stereotactic magnetic resonance-guided adaptive radiation therapy for localized kidney cancer: early outcomes from a prospective phase 1 trial and supplemental cohort. European Urology Oncology.

[47] Chang J.H., Cheung P., Erler D., Sonier M., Korol R., Chu W. (2016). Stereotactic ablative body radiotherapy for primary renal cell carcinoma in non-surgical candidates: initial clinical experience. Clin Oncol.

[48] Ponsky L., Lo S.S., Zhang Y., Schluchter M., Liu Y., Patel R. (2015). Phase I dose-escalation study of stereotactic body radiotherapy (SBRT) for poor surgical candidates with localized renal cell carcinoma. Radiother Oncol.

[49] Swaminath A., Cheung P., Glicksman R.M., Donovan E.K., Niglas M., Vesprini D. (2021). Patient-reported quality of life following stereotactic body radiation therapy for primary kidney cancer – results from a prospective cohort study. Clin Oncol.

[50] Grant S.R., Lei X., Hess K.R., Smith G.L., Matin S.F., Wood C.G. (2020). Stereotactic body radiation therapy for the definitive treatment of early stage kidney cancer: a survival comparison with surgery, tumor ablation, and observation. Adv Radiat Oncol.

[51] Michael S., Markus B., Boris S., Jozefina C., Alexander K., Alexander R. (2015). Single fraction radiosurgery for the treatment of renal tumors. J Urol.

[52] Rakshith H.T., Lohita S., Rebello A.P., Goudanavar P.S., Raghavendra N.N. (2023). Sex differences in drug effects and/or toxicity in oncology. Curr Res Pharmacol Drug Discovery.

[53] Davidson M., Wagner A.D., Kouvelakis K., Nanji H., Starling N., Chau I. (2019). Influence of sex on chemotherapy efficacy and toxicity in oesophagogastric cancer: a pooled analysis of four randomised trials. Eur J Cancer.

[54] Siegel RL, Miller KD, Wagle NS, Jemal A. Cancer statistics, 2023. CA: A Cancer Journal for Clinicians. 2023;73(1):17-48. 10.3322/caac.21763 36633525

[55] Nkemjika S., Tokede O., Okosun I.S., Jadotte Y., Pigott T. (2023). Biological sex disparity in survival outcomes following treatment for renal cell carcinoma: a systematic review and meta-analysis. Cancer Epidemiol.

[56] Kammula A.V., Schäffer A.A., Rajagopal P.S., Kurzrock R., Ruppin E. (2024). Outcome differences by sex in oncology clinical trials. Nat Commun.

[57] Sosinsky A.Z., Rich-Edwards J.W., Wiley A., Wright K., Spagnolo P.A., Joffe H. (2022). Enrollment of female participants in United States drug and device phase 1-3 clinical trials between 2016 and 2019. Contemp Clin Trials.

[58] Kidney Cancer Research: Sex-Inclusive but Sex-Unspecific. Clinical Oncology and Research. 2020.

[59] Kaneko S., Li X. (2018). X chromosome protects against bladder cancer in females via a KDM6A-dependent epigenetic mechanism. Sci Adv.

[60] Dunford A., Weinstock D.M., Savova V., Schumacher S.E., Cleary J.P., Yoda A. (2017). Tumor-suppressor genes that escape from X-inactivation contribute to cancer sex bias. Nat Genet.

[61] Büscheck F., Fraune C., Garmestani S., Simon R., Kluth M., Hube-Magg C. (2021). Y-chromosome loss is frequent in male renal tumors. Ann Transl Med.

[62] Brownmiller T., Juric J.A., Ivey A.D., Harvey B.M., Westemeier E.S., Winters M.T. (2020). Y chromosome LncRNA are involved in radiation response of male non-small cell lung cancer cells. Cancer Res.

[63] Abdel-Hafiz H.A., Schafer J.M., Chen X., Xiao T., Gauntner T.D., Li Z. (2023). Y chromosome loss in cancer drives growth by evasion of adaptive immunity. Nature.

[64] Gan X., Dai G., Li Y., Xu L., Liu G. (2024). Intricate roles of estrogen and estrogen receptors in digestive system cancers: a systematic review. Cancer Biol Med.

[65] Dakup P.P., Greer A.J., Gaddameedhi S. (2022). Let's talk about sex: a biological variable in immune response against melanoma. Pigment Cell Melanoma Res.

[66] Seydoux C., Briki M., Wagner A.D., Choong E., Guidi M., Carrara S. (2025). Importance of sex-dependent differences for dosing selection and optimization of chemotherapeutic drugs. Chemotherapy.

