# Supplementary material for: Biological sex representation and reporting in stereotactic body radiotherapy for kidney cancer: A review of clinical studies
Source: Clin Transl Radiat Oncol. 2025 Aug 18;55:101034. doi: 10.1016/j.ctro.2025.101034 (PMC12395976; doi:10.1016/j.ctro.2025.101034)
Supplement: Supplementary Data 1 [file mmc1.docx]

**SUPPLEMENTARY INFORMATION.** Full search strategy by online databases.

**EMBASE:**

'kidney cancer'/exp OR 'kidney tumor'/exp

((Kidney OR renal) NEAR/3 (cancer OR tumor* OR tumour* OR carcinoma* OR neoplasm*)):ti,ab,kw

#1 OR #2

'stereotactic body radiation therapy'/exp

('SABR' OR 'SABRT' OR 'SBRT'):ti,ab,kw

(stereotactic NEAR/3 ('radiation therapy' OR radiotherapy)):ti,ab,kw

#4 OR #5 OR #6

'clinical trial'/de OR 'randomized controlled trial'/de OR 'randomization'/de OR 'single blind procedure'/de OR 'double blind procedure'/de OR 'crossover procedure'/de OR 'placebo'/de OR 'prospective study'/de OR ('randomi?ed controlled' NEXT/1 trial*) OR rct OR 'randomly allocated' OR 'allocated randomly' OR 'random allocation' OR (allocated NEAR/2 random) OR (single NEXT/1 blind*) OR (double NEXT/1 blind*) OR ((treble OR triple) NEAR/1 blind*) OR placebo*

#3 AND #7 AND #8

**Web of Science (all databases):**

((Kidney OR renal) NEAR/3 (cancer OR tumor* OR tumour* OR carcinoma* OR neoplasm*)) AND (SABR OR SABRT OR SBRT OR (stereotactic NEAR/3 ("radiation therapy" OR radiotherapy))) AND ("clinical trial*" OR "research design" OR "comparative stud*" OR "evaluation stud*" OR "controlled trial*" OR "follow-up stud*" OR "prospective stud*" OR random* OR placebo* OR "single blind*" OR "double blind*")

**CINAHL:**

(TI ((Kidney OR renal) N3 (cancer OR tumor* OR tumour* OR carcinoma* OR neoplasm*)) AND (SABR OR SABRT OR SBRT OR (stereotactic N3 ("radiation therapy" OR radiotherapy))) OR AB ((Kidney OR renal) N3 (cancer OR tumor* OR tumour* OR carcinoma* OR neoplasm*)) AND (SABR OR SABRT OR SBRT OR (stereotactic N3 ("radiation therapy" OR radiotherapy)))) AND (TX allocat* random* OR (MH "Quantitative Studies") OR (MH "Placebos") OR TX placebo* OR TX random* allocat* OR (MH "Random Assignment") OR TX randomi* control* trial* OR TX ( (singl* n1 blind*) OR (singl* n1 mask*) ) OR TX ( (doubl* n1 blind*) OR (doubl* n1 mask*) ) OR TX ( (tripl* n1 blind*) OR (tripl* n1 mask*) ) OR TX ( (trebl* n1 blind*) OR (trebl* n1 mask*) ) OR TX clinic* n1 trial* OR PT Clinical trial OR (MH "Clinical Trials+"))
